# Variable pressure support ventilation vs. biphasic positive airway pressure/airway pressure release ventilation in experimental acute respiratory failure

**DOI:** 10.1186/s12871-026-04228-6

**Published:** 2026-09-15

**Authors:** Thomas Bluth, Maren Thiel, Dirk Haufe, Thomas Kiss, Thea Koch, Marcelo Gama de Abreu, Peter Markus Spieth, Andreas Güldner

**Affiliations:** 1 Department of Anaesthesiology and Intensive Care Medicine, Pulmonary Engineering Group, Faculty of Medicine, University Hospital Carl Gustav Carus, TUD Dresden University of Technology, Fetscherstraße 74, Dresden, 01307 Germany; 2 AMD TÜV Occupational Health Services Ltd, TÜV Rheinland Group, Berlin, Germany; 3 Department of Anesthesiology, Intensive-, Pain- and Palliative Care Medicine, Radebeul Hospital, Academic Hospital of the Technische Universität Dresden, Radebeul, Germany; 4 Division of Surgical Critical Care, Department of Anesthesiology, Integrated Hospital Care Institute, Cleveland Clinic, Cleveland, OH USA; 5 Division of Cardiothoracic Anesthesia, Department of Anesthesiology, Integrated Hospital Care Institute, Cleveland Clinic, Cleveland, OH USA; 6 Department of Anesthesiology, Outcomes Research Consortium, Integrated Hospital Care Institute, Cleveland, OH USA

**Keywords:** Mechanical ventilation, BIPAP, Variable ventilation, Randomized trial, Animal, lung injury

## Abstract

**Background:**

Spontaneous breathing during mechanical ventilation (MV) can improve cardiorespiratory function and lung protection. Biphasic Positive Airway Pressure (BIPAP) and Airway Pressure Release Ventilation (APRV) are common MV modes that combine spontaneous breathing with controlled ventilation. Variable pressure support ventilation (PSV), which varies breath-by-breath pressure support randomly, has been shown to improve gas exchange and reduce lung injury compared to conventional MV modes. This study aimed to compare the short-term effects of variable PSV with BIPAP in a model of acute respiratory failure, hypothesizing that variable PSV would improve lung function without increasing lung damage or inflammation.

**Methods:**

In an exploratory randomized study with 18 pigs, lung injury was induced by lung lavage. Over 4 h, two lung protective strategies were applied with individually optimized PEEP: (1) BIPAP with non-assisted spontaneous breathing and (2) variable PSV, maintaining a mean tidal volume of 6 ml/kg body weight while varying pressure support. Plasma and bronchoalveolar lavage fluid samples were collected for inflammation markers, and lung damage was assessed histologically. Gene expression of inflammatory cytokines was also measured.

**Results:**

Variable PSV resulted in slightly improved gas exchange with higher mean tidal volumes and minute ventilation compared to BIPAP, as well as reduced work of breathing. Histopathological analysis showed alveolar damage to be mild, with no significant overall difference between the groups. However, variable PSV caused more alveolar edema, especially in gravity-dependent lung regions, and increased the wet-to-dry ratio compared with BIPAP. Despite these differences, there were no significant changes in systemic or pulmonary inflammatory cytokines or gene expression between the two groups.

**Conclusion:**

In this model of acute respiratory failure, variable PSV with a protective tidal volume of 6 ml/kg resulted in marginal, but clinically insignificant improvements in gas exchange and reduced work of breathing compared to BIPAP with non-assisted spontaneous breathing. Although inflammatory markers and overall diffuse alveolar damage did not differ between ventilation modes, the findings suggest a possible association between variable PSV and increased pulmonary oedema compared with BIPAP.

## Background

Maintaining or restoring spontaneous breathing early during mechanical ventilation in patients suffering from acute respiratory failure can improve gas exchange and respiratory mechanics, reduce the need for sedatives and cardiovascular support and is finally able to reduce the total time of mechanical ventilation [1–4]. Reduced time of ventilatory support may reduce the risk of ventilator induced diaphragmatic dysfunction [5] and the development of other ventilator associated complications.

Another approach to improving mechanical ventilation is to continuously vary mandatory ventilation parameters. It has been shown that variable tidal volumes can improve gas exchange and respiratory mechanics in different experimental settings [6–13]. Based on some of these initial findings variable pressure support ventilation (variable PSV) had been developed [14], which theoretically combines the positive effects of spontaneous breathing modes and the restored physiological breathing pattern variability. Experimental evidence suggests that application of extrinsic variability in pressure support as with variable PSV is associated with beneficial effects in experimental lung injury [9, 11, 14, 15], and was well tolerated during short term application in patients with acute respiratory failure [16].

Most studies showing positive effects of spontaneous breathing in acute respiratory distress syndrome (ARDS) were performed with biphasic positive airway pressure / airway pressure release ventilation (BIPAP/APRV), favoring BIPAP/APRV as the standard ventilatory mode for maintenance of spontaneous breathing [2].

The present study based on findings that BIPAP and PSV are associated with distinct short term effects on pulmonary aeration and perfusion [17, 18]. During PSV, spontaneous inspiratory effort is consistently augmented by ventilator-delivered pressure support, potentially increasing mechanical load on lung tissue. In contrast, spontaneous breaths during BIPAP/APRV may remain unsupported, which could reduce lung stress while preserving the benefits of spontaneous breathing. Consequently, it remains unclear whether the improved ventilatory assistance provided by variable PSV translates into superior lung function or whether it comes at the expense of reduced lung protection.

To address this question, we compared variable PSV with BIPAP/APRV in a porcine model of surfactant depletion-induced acute respiratory failure. Both modes were implemented to reflect routine clinical practice, allowing straightforward switching between ventilation strategies while maintaining comparable sedation, PEEP, and protective tidal volumes. We hypothesized that the application of variable pressure support improves gas exchange and respiratory mechanics without causing increased pulmonary inflammatory response.

## Methods

### Ethics and consent to participate

The institutional animal care and welfare committee and the government of the state of Saxony, Germany, approved all animal procedures following federal law (Landesdirektion Dresden, Germany, File AZ 24D-9168.11-1/2006-18). Animals were purchased from a private owner (Agrar Service GmbH Schweinemast Horka, Germany). This company provided an informed consent statement to use the animals in the current study. Animals received humane care in compliance with the Principles of Laboratory Animal Care formulated by the National Society for Medical Research and the US National Academy of Sciences Guide for the Care and Use of Laboratory Animals and reported according to the Animal Research: Reporting In Vivo Experiments statements.

### Trial design and primary endpoint

This was an explorative randomized controlled experimental study. For structure and clarity of the manuscript, a primary endpoint was defined, which was the ratio between arterial partial pressure of oxygen and fraction of inspired oxygen (PaO_2_/FiO2, Horovitz index). Relevant secondary endpoints included parameters of respiratory mechanics, as well as systemic and pulmonary inflammation and alveolar damage.

### Animal preparation and mechanical ventilation settings

Eighteen juvenile pigs (22–30 kg; German landrace) were intramuscularly premedicated with ketamine (10 mg/kg) and midazolam (1 mg/kg). Anesthesia was induced by intravenous boli of ketamine and midazolam and maintained by continuous infusion (12–18 mg/kg/h ketamine and 2 mg/kg/h midazolam). Muscle paralysis was achieved by infusion of atracurium (1 mg/kg/h). Animals were orotracheally intubated with an 8.0 ID endotracheal tube and ventilated in volume controlled mode using an EVITA XL ventilator (Dräger Medical, Lübeck, Germany). Initial ventilator settings were as follows: tidal volume (V_T_) 10 ml/kg of actual body weight, FiO_2_ 1.0, positive endexpiratory pressure (PEEP) 5 cmH_2_O, inspiratory: expiratory ratio (I: E) 1:1 and respiratory rate to achieve arterial carbon dioxide (PaCO_2_) of 35–45 mmHg at fixed inspiratory flow of 35 l/min. Fluid uptake was maintained by infusion of a balanced crystalloid solution (10 ml/kg/h). After right lateral neck incision a 5 Fr. catheter was placed into the internal carotid artery and a 7.5 Fr. Swan-Ganz catheter was advanced via an 8.5 Fr. sheath into the pulmonal artery. Urine drainage was performed by invasive catheterization of the urinary bladder via mini laparotomy. Animals were kept in supine position during the entire experiment.

Experimental respiratory failure was induced by repetitive saline lung lavage (30 ml/kg) in supine position until PaO_2_/FiO_2_ fell constantly below 200 mmHg for 30 min [19].

Following injury, lung recruitment by means of a continuous pressure of 50 cmH_2_O during 30 s and a consecutive decremental PEEP trial was conducted in order to set PEEP according to optimal respiratory system mechanics as described earlier [20]. Briefly, PEEP was set at 20 cmH_2_O and reduced in steps of 2 cmH_2_O after each 2 min while keeping other respiratory parameters unchanged. After this, lung recruitment was repeated, and PEEP was set at the level corresponding to the minimal elastance of the respiratory system (E_RS_). Prior to resuming of spontaneous breathing, atracurium was stopped and ketamine and midazolam infusion was reduced.

Animals were randomly assigned to variable PSV or BIPAP. During BIPAP spontaneous breathing was facilitated and considered sufficient, if the amount of minute ventilation (MV) of spontaneous breaths reached minimum 20% of total MV according to the algorithm implemented in the ventilator. In order to apply unassisted spontaneous breathing and therefore avoid synchronization of the pressure changes and the subject’s effort, as implemented in BiPAP ventilation mode, BIPAP was performed using the APRV mode embedded in the ventilator. However, because APRV is commonly regarded in clinical practice as a mode employing inverse ratio ventilation, the term BIPAP is used in this study to clarify the ventilatory settings applied. Variable PSV was applied via external remote control of the ventilator as previously described [14]. Ventilator settings are summarized in Table 1. Assisted tidal volumes during variable PSV and mandatory (controlled) tidal volumes during BIPAP, resulting from the switch between PEEP and inspiratory airway pressure, were matched between groups.

**Table 1:** Ventilator settings in experimental groups

Ventilator settings	BIPAP	Variable PSV
FiO_2_	1.0	1.0
PEEP	according to individual optimum PEEP (minimal E_RS_)	according to individual optimum PEEP (minimal E_RS_)
I: E	1:1	approx. 1:4
V_T_	target 6 ml/kg BW for controlled beaths; additional spontaneous breaths remained without mechanical assistance	mean target 6 ml/kg BW
Respiratory rate	15/min controlled	spontaneous
T_high_	2s	spontaneous
T_low_	2s	N/A
P_high_	to reach V_T_ of 6 ml/kg	N/A
P_low_	according to individual optimum PEEP (minimal E_RS_)	N/A
Ramp	0.2 s	N/A
P_ASB_	N/A	to reach V_T_ of 6 ml/kg
Cycling off	N/A	25% of peak flow
Flow trigger	N/A	2.0 l/min

*BIPAP *biphasic positive airway pressure. *PSV *pressure support ventilation. *FiO*_2_ fraction of inspired oxygen. *PEEP* positive endexpiratory pressure. I: E, inspiratory: expiratory ratio. *V*_T_ tidal volume. *T*_high_, time on upper pressure level. *T*_low_, time on low pressure level. *P*_high_, pressure of upper pressure level. *P*_low_, pressure of lower pressure level. *Ramp* pressure rise time. *P*_ASB_ pressure of spontaneous breath assistance. *BW *body weight. E_RS_, elastance of the respiratory system

After 4 h of study intervention had been completed, animals were sacrificed by intravenous injection of 1 M-potassium chloride (50 ml) following deepening of general anesthesia with thiopental (2 g). The lungs were removed at continuous airway pressures equal to PEEP. After separation, the middle lobe of the right lung was weighed (wet weight) and then rinsed two times with 50 ml with saline solution to obtain bronchoalveolar lavage fluid (BALF). After standardized drying by microwave heating the middle lobe was weighed again (dry weight), and the ratio between wet and dry weight calculated (wet-to-dry ratio) [21]. Tissue samples were taken from dependent and nondependent areas of both lungs according to a fixed protocol.

### Data acquisition and measurements

Airway flow was measured using a heated pneumotachograph (Fleisch No. 2; Fleisch, Lausanne, Switzerland) connected to a differential pressure transducer (PXL12 × 5DN; Sensortechnics, Troy, NY) and airway pressures were obtained using a pressure transducer (SCX01DNC; SenSym ICT, Milpitas, CA.) at the endotracheal tube. After advancing an esophageal balloon catheter (Erich Jaeger, Höchberg, Germany) into mid-chest level, esophageal pressure was measured by a third pressure transducer (SCX01DNC, SenSym ICT). The signals of airway pressure, esophageal pressure, and airway flow were recorded using a LabView (National Instruments, Austin, TX) based data acquisition system as described previously [20].

Continuous recordings (5 min) of respiratory mechanics, gas exchange, hemodynamics and blood samples for plasma cytokine analysis were assessed at baseline, injury, after titration of individual PEEP and resuming of spontaneous breathing (baseline 2) and 1-hour-intervals during the study intervention thereafter (Time 1 to 4). Parameters of respiratory mechanics were calculated offline as described elsewhere [20]. Airway pressure at 100 ms after beginning of inspiration (P_0.1_) was determined and used as surrogate of the central respiratory drive. Transpulmonary right-to-left shunt was calculated using standard formula, as previously described [20]. Using the recorded respiratory signals, tidal volumes were semiautomatically (algorithm-based detection and visually supervision of correct identification) classified as mandatory, assisted spontaneous and non-assisted spontaneous.

Scoring of Diffuse alveolar damage, which includes typical histopathological features of the early exudative phase of ARDS, was performed in tissue samples from gravity dependent and non-dependent regions of the left lung as described before [22]. mRNA expression of Interleukin (IL)-6, IL-8 and Amphiregulin in right lung tissue samples was analyzed using quantitative real-time polymerase chain reaction as previously described [10]. Protein levels of IL-6 and IL-8 were measured in plasma at all time points and in BALF supernatant using commercially available enzyme linked immunosorbent assay kits according to the manufacturer’s instructions.

### Statistical analysis

Data are presented as mean ± standard deviation unless stated otherwise. Student´s t-test, one-way analysis of variance or repeated measures analysis of variance were used for normal distributed data as appropriate, Mann-Whitney-U-test and Wilcoxon-test were used in case of non-normal distributed data or nominal scaled variables. All tests were performed using the SPSS software package (Vers. 15.0, SPSS Inc., Chicago, IL). Multiple comparisons were adjusted according to Sidak or Bonferroni-Holm procedure as appropriate. The global significance level was defined as *P* < .05 and, despite the exploratory nature of the study, is indicated in the text and tables for improved clarity.

## Results

**Fig. 1:**
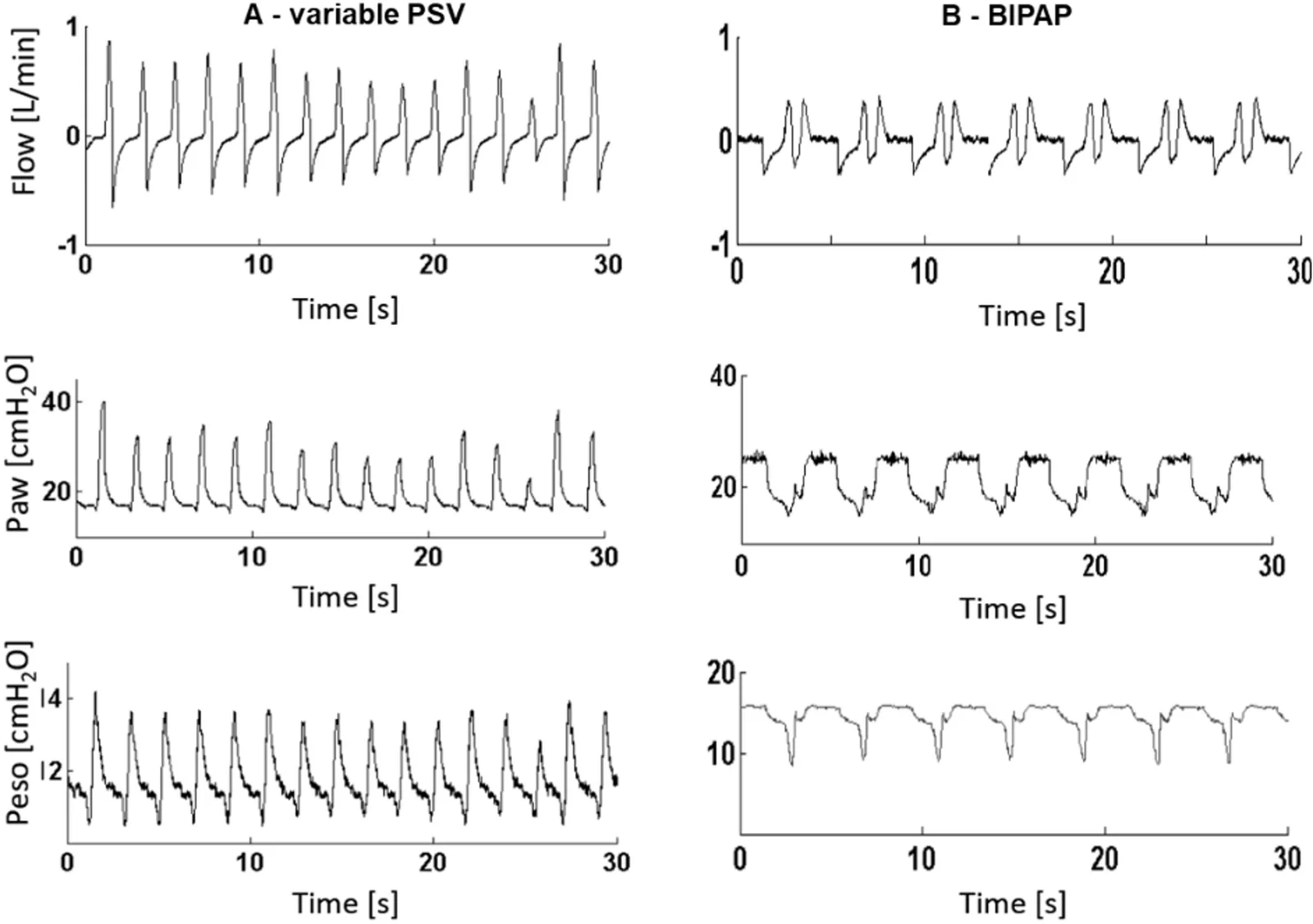
– Typical Recordings. Typical recordings of airway flow (Flow), airway (P_aw_) and esophageal (P_eso_) pressure over time. Variable PSV (Panel **A**) = variable Pressure Support Ventilation; BIPAP (Panel **B**) biphasic positive airway pressure ventilation.

Groups did not differ significantly regarding bodyweight (mean [min-max]; variable PSV 27 [25–31]; BIPAP 27 [22–30]; *p* ≥ .05) and number of lavages (mean [min-max]; variable PSV 8 [2–14]; BIPAP 8 [1–12]; *p* ≥ .05). Typical recordings of airway flow, airway and esophageal pressure are depicted in Fig. 1.

### Gas exchange

**Fig. 2:**
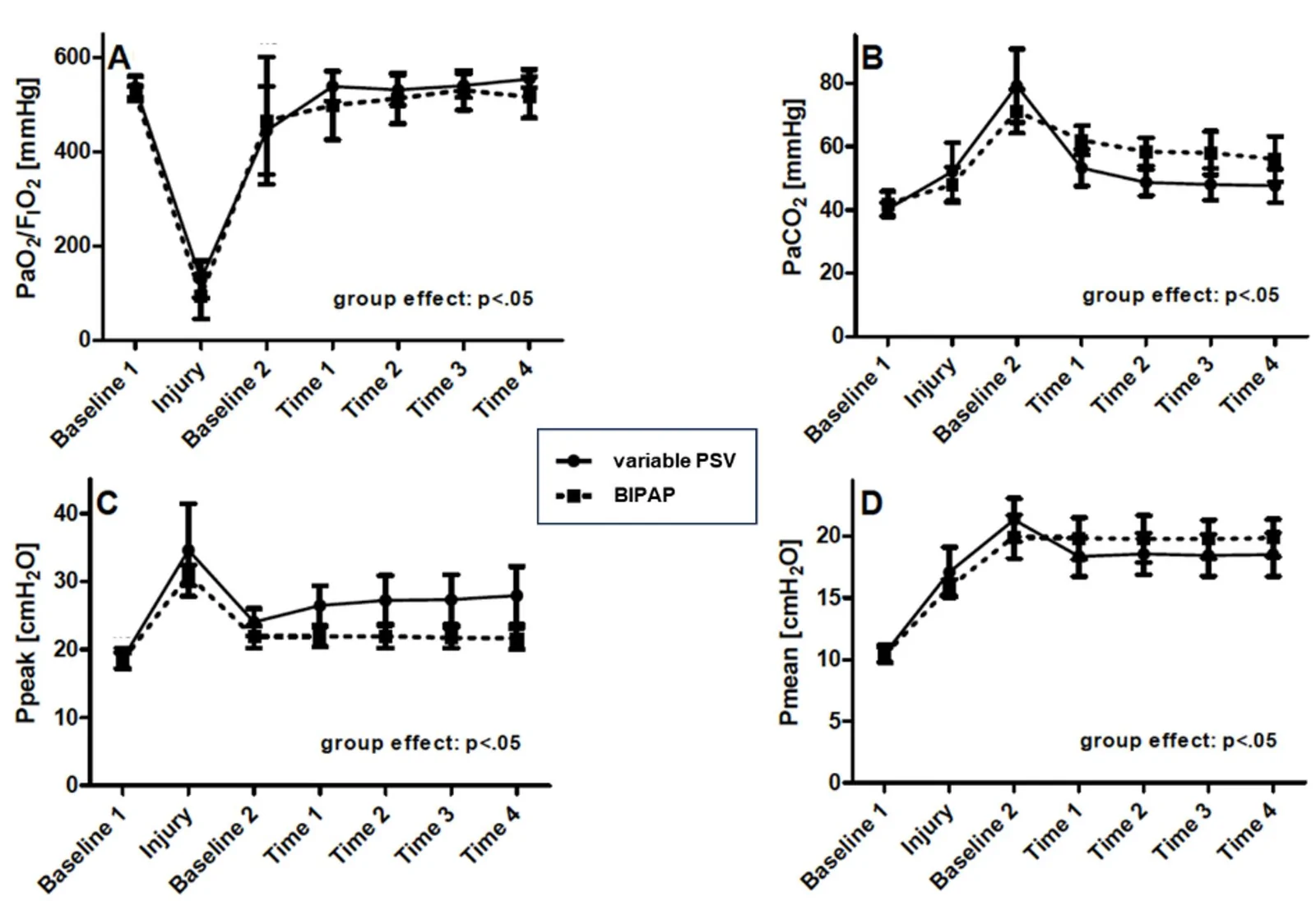
Gas Exchange and Airway Pressures. Ratio of arterial partial pressure of oxygen and inspired oxygen fraction (PaO_2_/F_i_O_2_ – Panel **A**); arterial partial pressure of carbon dioxide (PaCO_2_ – Panel **B**); peak airway pressure (P_peak_ – Panel **C**) and mean airway pressure (P_mean_ – Panel **D**). Variable PSV = variable Pressure Support Ventilation; BIPAP biphasic positive airway pressure ventilation

Data on gas exchange are presented in Fig. 2; Table 2. Induction of lung injury significantly decreased PaO_2_/FiO_2_ and pH and increased PaCO_2_ and Shunt in both groups (Baseline 1 vs. Injury). Optimized PEEP and resuming of spontaneous breathing (Injury vs. Baseline 2) led to significantly increased PaO_2_/FiO_2_ and PaCO_2_ and decreased pH and Shunt. The primary endpoint, PaO_2_/FiO_2_, and pH were significantly increased with variable PSV as compared to BIPAP. PaCO_2_ were significantly lower with variable PSV as compared to BIPAP, whereas Shunt did not differ significantly between groups.

**Table 2:** Gas exchange and hemodynamics

Variable	Group	Baseline 1	Injury	Baseline 2	Time 1	Time 2	Time 3	Time 4	Statistics (group effect)
pH	*variable PSV*	7.43 ± 0.05	7.32 ± 0.08	7.21 ± 0.05	7.37 ± 0.05	7.40 ± 0.06	7.42 ± 0.05	7.42 ± 0.05	*p*<.05*^†^
	*BIPAP*	7.40 ± 0.05	7.34 ± 0.05	7.23 ± 0.04	7.28 ± 0.04	7.32 ± 0.04	7.34 ± 0.05	7.35 ± 0.06	
venous admixture (%)	*variable PSV*	11.1 ± 3.1	24.3 ± 5.1	10.7 ± 5.0	5.6 ± 1.6	6.4 ± 2.3	5.6 ± 1.7	4.8 ± 1.0	ns*^†^
	*BIPAP*	15.3 ± 5.3	34.5 ± 13.1	7.4 ± 1.8	7.4 ± 2.1	7.3 ± 2.1	5.7 ± 2.1	6.4 ± 0.9	
HR (min^− 1^)	*variable PSV*	96 ± 12	78 ± 19	82 ± 18	69 ± 1	72 ± 10	71 ± 8	70 ± 8	ns*
	*BIPAP*	104 ± 17	82 ± 12	72 ± 8	71 ± 9	69 ± 1	71 ± 11	68 ± 9	
MAP (mmHg)	*variable PSV*	79 ± 1	77 ± 1	71 ± 23	78 ± 2	80 ± 1	82 ± 10	81 ± 12	ns
	*BIPAP*	73 ± 11	79 ± 13	73 ± 13	76 ± 14	77 ± 12	79 ± 14	77 ± 12	
MPAP (mmHg)	*variable PSV*	26 ± 3	36 ± 4	32 ± 4	30 ± 3	30 ± 3	29 ± 5	30 ± 3	ns*^†^
	*BIPAP*	25 ± 2	31 ± 4	29 ± 5	30 ± 4	29 ± 4	30 ± 3	30 ± 3	
CO (l min^− 1^)	*variable PSV*	3.7 ± 1.1	2.6 ± 0.8	2.8 ± 0.5	2.2 ± 0.3	2.3 ± 0.4	2.3 ± 0.4	2.2 ± 0.4	*p*<.05*
	*BIPAP*	4.4 ± 1.1	3.2 ± 0.8	2.6 ± 0.4	2.7 ± 0.6	2.5 ± 0.5	2.3 ± 0.5	2.3 ± 0.3	
PAOP (mmHg)	*variable PSV*	17 ± 1	17 ± 1	19 ± 1	18 ± 1	18 ± 1	18 ± 2	18 ± 1	ns^†^
	*BIPAP*	17 ± 2	16 ± 2	18 ± 2	18 ± 2	18 ± 2	18 ± 2	17 ± 1	
SVR (dyn s cm^− 5^)	*variable PSV*	1536 ± 691	1725 ± 509	1700 ± 270	2217 ± 332	2264 ± 353	2305 ± 475	2321 ± 423	*p*<.05
	*BIPAP*	1176 ± 507	1676 ± 521	1769 ± 330	1802 ± 337	1925 ± 313	2118 ± 264	2136 ± 259	
PVR (dyn s cm^− 5^)	*variable PSV*	228 ± 134	522 ± 109	388 ± 117	437 ± 148	454 ± 146	445 ± 119	464 ± 165	ns*
	*BIPAP*	208 ± 167	392 ± 82	372 ± 121	366 ± 106	349 ± 81	416 ± 67	440 ± 80	

Data are mean ± standard deviation. *PSV *pressure support ventilation. *BIPAP *biphasic positive airway pressure. venous admixture, transpulmonary right-to left shunt calculated according to standard formula. HR, heart rate. *MAP *mean arterial pressure. *MPAP *mean pulmonary arterial pressure. *CO *cardiac output. *PAOP *pulmonary artery occlusion pressure. *SVR *systemic vascular resistance. *PVR *pulmonary vascular resistance. *N/A *not applicable. ns, non-significant. Effects of intervention (Statistics; group effect) were assessed by general linear model statistics and corrected for multiple comparisons by the Sidak procedure. *p*<.05 was considered statistically significant. Differences between Baseline 1 and Injury as well as Injury and Baseline 2 within the animals were tested using t-tests for dependent samples and indicated in footnotes: *p < .05 for Baseline versus Injury; †*p* < .05 for Injury versus Baseline 2

### Respiratory variables

**Fig. 3:**
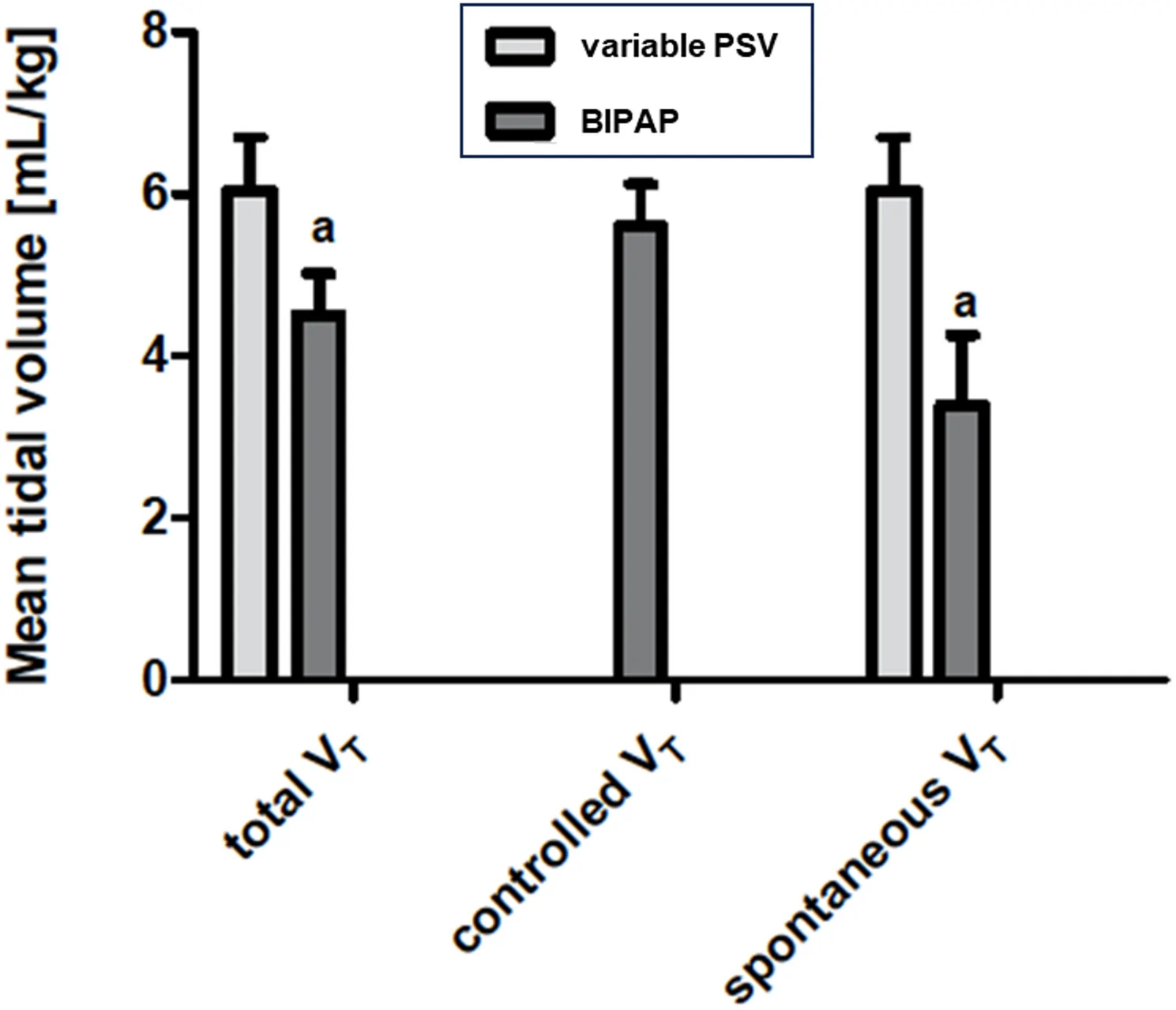
Mean tidal volumes during study intervention. Variable PSV = variable Pressure Support Ventilation; BIPAP biphasic positive airway pressure ventilation. V_T_ = tidal volume. a = *p*<.05 vs. variable PSV

Respiratory data are presented in Figs. 2; Table 3. Lung injury led to increased peak and mean airway pressures (P_peak_, P_mean_) without affecting other respiratory variables. Individually adjusted PEEP level did not relevantly differ in both groups (variable PSV 16 ± 1; BIPAP 15 ± 2 cmH_2_O; *p* ≥ .05). Reduction of tidal volumes during resuming of spontaneous breathing was associated with a consecutive decrease in minute ventilation and increased respiratory rate as well as increased, but not relevant intrinsic PEEP. Total and spontaneous tidal volume and minute ventilation were significantly increased with variable PSV compared to BIPAP (Fig. 3). Total respiratory rate did not differ significantly between groups, but spontaneous respiratory rate was significantly increased with variable PSV. P_peak_ was higher and P_mean_ and pressure time product (PTP) were lower with variable PSV as compared to BIPAP. P_0.1_ was significantly lower with variable PSV compared to BIPAP.

**Table 3:** Lung mechanics

Variable	Group	Baseline 1	Injury	Baseline 2	Time 1	Time 2	Time 3	Time 4	Statistics (group effect)
MV _tot_ (L min^− 1^)	*variable PSV*	3.9 ± 0.6	3.9 ± 0.6	3.1 ± 0.6	4.7 ± 1.0	5.1 ± 1.0	4.8 ± 1.1	4.8 ± 1.1	*p*<.05^†^
	*BIPAP*	3.6 ± 0.4	3.7 ± 0.7	2.9 ± 0.4	3.6 ± 0.5	3.5 ± 0.6	3.6 ± 0.5	3.8 ± 0.5	
MV _spont_ (L min^− 1^)	*variable PSV*	N/A	N/A	0.8 ± 0.7	4.7 ± 1.0	5.1 ± 1.0	4.8 ± 1.1	4.8 ± 1.1	*p*<.05
	*BIPAP*	N/A	N/A	0.7 ± 0.4	1.3 ± 0.4	1.3 ± 0.4	1.4 ± 0.4	1.5 ± 0.4	
V_T tot_ (mL kg^− 1^)	*variable PSV*	9.8 ± 0.3	9.7 ± 0.2	4.4 ± 1.0	6.0 ± 0.7	6.2 ± 0.6	6.0 ± 0.8	6.1 ± 0.8	*p*<.05^†^
	*BIPAP*	9.8 ± 0.4	9.8 ± 0.5	4.2 ± 0.6	4.6 ± 0.9	4.6 ± 0.7	4.4 ± 0.6	4.4 ± 0.5	
V_T spont_ (mL kg^− 1^)	*variable PSV*	N/A	N/A	3.1 ± 1.6	6.0 ± 0.7	6.2 ± 0.6	6.0 ± 0.8	6.1 ± 0.8	*p*<.05
	*BIPAP*	N/A	N/A	2.1 ± 0.7	3.4 ± 1.2	3.4 ± 1.1	3.4 ± 0.9	3.4 ± 0.8	
RR _tot_ (min^− 1^)	*variable PSV*	15 ± 2	15 ± 3	26 ± 5	29 ± 5	30 ± 6	30 ± 7	29 ± 6	ns^†^
	*BIPAP*	14 ± 2	14 ± 3	27 ± 4	29 ± 1	29 ± 2	30 ± 3	32 ± 5	
RR _spont_ (min^− 1^)	*variable PSV*	N/A	N/A	10 ± 6	29 ± 5	30 ± 6	30 ± 7	29 ± 6	*p*<.05
	*BIPAP*	N/A	N/A	12 ± 4	14 ± 1	14 ± 2	15 ± 3	17 ± 5	
PEEP_i_ (cmH_2_O)	*variable PSV*	0.3 ± 0.3	0.4 ± 0.2	0.9 ± 0.5	0.7 ± 0.5	0.6 ± 0.4	0.6 ± 0.2	0.6 ± 0.2	ns^†^
	*BIPAP*	0.3 ± 0.2	0.3 ± 0.2	0.4 ± 0.2	0.7 ± 0.6	0.6 ± 0.4	0.5 ± 0.1	0.5 ± 0.1	
PTP (cmH_2_O∙s)	*variable PSV*	N/A	N/A	31.2 ± 25.2	21.4 ± 24.1	27.2 ± 35.9	25.7 ± 43.1	24.4 ± 44.9	*p*<.05
	*BIPAP*	N/A	N/A	24.3 ± 9.2	33.3 ± 30.2	35.3 ± 23.6	36.4 ± 15.5	38.1 ± 18.1	
P_0.1_ (cmH_2_O)	*variable PSV*	N/A	N/A	1.6 ± 0.8	0.5 ± 0.4	0.7 ± 0.6	0.6 ± 0.7	0.6 ± 0.7	*p*<.05
	*BIPAP*	N/A	N/A	1.3 ± 0.5	1.8 ± 0.8	1.8 ± 0.7	2.0 ± 0.6	2.0 ± 0.8	

Data are mean ± standard deviation. *PSV* pressure support ventilation. *BIPAP *biphasic positive airway pressure. *MV*_tot_*/MV*_spont_ minute ventilation including all (total)/spontaneous respiratory cycles. *V*_T tot_*/V*_T spont_ tidal volume including all (total)/spontaneous respiratory cycles. *RR*_tot_*/RR*_spont_ respiratory rate including all (total)/spontaneous respiratory cycles. *PEEP*_i_, intrinsic PEEP measured by endexpiratory hold. *PTP *pressure time product (cumulative pressure over time). P_0.1_, spontaneous inspiratory effort (pressure) at 0.1 s after start of inspiration. *N/A *not applicable. *ns *non-significant. Effects of intervention (Statistics; group effect) were assessed by general linear model statistics and corrected for multiple comparisons by the Sidak procedure. *p*<.05 was considered statistically significant. Differences between Baseline 1 and Injury as well as Injury and Baseline 2 within the animals were tested using t-tests for dependent samples and indicated in footnotes: *p < .05 for Baseline versus Injury; †*p* < .05 for Injury versus Baseline 2

### Hemodynamics

Hemodynamic data are shown in Table 2. Mean pulmonary arterial pressure (MPAP) and pulmonary vascular resistance increased with lung injury, whereas cardiac output decreased mainly by a reduced heart rate. Optimized PEEP and induction of spontaneous breathing led to a significant decrease of MPAP. Analysis of differences between groups revealed decreased cardiac output and increased systemic vascular resistance with variable PSV compared to BIPAP, whereas other variables did not differ.

### Histology and lung edema

Cumulative alveolar damage scores did not differ significantly between the experimental groups, although lungs ventilated with noisy PSV showed significantly more alveolar oedema, particularly in gravity-dependent regions (Table 4). Other characteristics of the score showed no significant differences between the groups. Independent of group allocation, alveolar overdistension was more pronounced in non-dependent compared to dependent regions and achieved the highest scores among all features. The wet-to-dry weight ratio were higher in lungs ventilated with variable PSV compared to BIPAP (8.0 ± 0.8 versus 7.1 ± 0.4; *p* < .05).

**Table 4:** Diffuse alveolar damage score

	**group**	**alveolar edema**	**interstitial edema**	**hemorrhage**	**inflammatory infiltration**	**epithelial destruction**	**microatelectasis**	**overdistension**	**cumulated Score**
ventral (non-dependent)	**variable PSV**	2 [2–3]	3 [3–4]	1 [0–2]	4 [4–5]	5 [4–5]	3 [2–3]	28 [24–30]	49 [43–50]
	**BIPAP**	2 [1–3]	3 [2–3]	0 [0–2]	3 [2–6]	4 [3–5]	3 [2–3]	18 [14–25]	35 [21–43]
		ns	ns	ns	ns	ns	ns	ns	ns
dorsal (dependent)	**variable PSV**	3 [3–4]	4 [2–5]	4 [0–4]	6 [4–7]	5 [3–6]	2 [2–3]	18 [12–20]	41 [38–44]
	**BIPAP**	2 [1–2]	4 [2–4]	2 [0–2]	4 [2–8]	4 [4–6]	3 [2–4]	10 [9–20]	34 [22–42]
		*p*<.05	ns	ns	ns	ns	ns	ns	ns
overall	**variable PSV**	3 [2–4]	3 [2–4]	1 [0–4]	5 [4–7]	5 [3–6]	3 [2–3]	22 [16–28]	43 [38–50]
	**BIPAP**	2 [1–3]	3 [2–4]	2 [0–2]	3 [2–7]	4 [3–6]	3 [2–3]	18 [9–24]	35 [21–43]
		*p*<.05	ns	ns	ns	ns	ns	ns	ns

Data are median and interquartile. Cumulated score represents addition of single score characteristics. *PSV *pressure support ventilation. *BIPAP *biphasic positive airway pressure. ns, non-significant. Statistical tests among groups were performed using the Mann Whitney U Test. *p*<.05 was considered statistically significant

### Biomarkers of lung injury

mRNA expression of IL-6, IL-8 and Amphiregulin did not differ significantly between groups, independent from lung region (Table 5). Protein levels of IL-6 and IL-8 in plasma did not differ significantly between groups at any time point (Table 6), nor did IL-6 (variable PSV 877 [213–1712]; BIPAP 356 [232–661]; *p* ≥ .05) and IL-8 (variable PSV 170 [116–272]; BIPAP 99 [97–198]; *p* ≥ .05) in bronchoalveolar lavage fluid.

**Table 5:** Pulmonary inflammation – x-fold mRNA expression in lung tissue

	**group**	**IL-6**	**IL-8**	**Amphiregulin**
ventral (non-dependent)	variable PSV	0.04 [0.02 − 0.11]	1.05 [0.54–1.23]	2.14 [1.33–2.76]
	BIPAP	0.03 [0.02 − 0.04]	0.80 [0.56 − 0.95]	2.03 [1.49–2.41]
		ns	ns	ns
dorsal (dependent)	variable PSV	1.01 [0.35–4.53]	0.97 [0.27–1.04]	1.59 [1.21–3.75]
	BIPAP	0.46 [0.24 − 0.55]	0.87 [0.38–1.24]	3.62 [1.47–7.22]
		ns	ns	ns
overall	variable PSV	0.23 [0.04–1.03]	0.99 [0.43–1.23]	1.91 [1.21–2.96]
	BIPAP	0.09 [0.03 − 0.46]	0.80 [0.46–1.12]	2.19 [1.49–3.79]
		ns	ns	ns

Data are median and interquartile. Genes were normalized to expression of β-2-microglobulin (housekeeping gene). *PSV *pressure support ventilation. *BIPAP *biphasic positive airway pressure. *IL *interleukin. ns, non-significant. Statistical tests among groups were performed using the Mann Whitney U Test. *p*<.05 was considered statistically significant

**Table 6:** Systemic inflammation - protein (pg/ml) in plasma

	**group**	**Baseline 1**	**Injury**	**Baseline 2**	**Time 1**	**Time 2**	**Time 3**	**Time 4**	**Group effect**
IL 6	**variable PSV**	0.16 ± 0.15	0.13 ± 0.09	0.11 ± 0.04	0.35 ± 0.71	0.12 ± 0.05	0.11 ± 0.04	0.12 ± 0.04	ns
	**BIPAP**	0.12 ± 0.05	0.14 ± 0.05	0.12 ± 0.05	0.12 ± 0.03	0.14 ± 0.05	0.18 ± 0.13	0.15 ± 0.08	
IL 8	**variable PSV**	1.71 ± 3.78	0.66 ± 0.86	1.18 ± 2.27	0.87 ± 0.99	1.23 ± 1.61	1.12 ± 1.41	1.16 ± 1.58	ns
	**BIPAP**	3.07 ± 3.74	5.14 ± 6.48	2.49 ± 2.48	3.49 ± 4.88	2.20 ± 2.61	2.69 ± 3.13	2.41 ± 3.01	

Data are mean ± standard deviation. *PSV *pressure support ventilation. *BIPAP *biphasic positive airway pressure. *IL *interleukin. ns, non-significant. Effects of therapy (group effect) were assessed by general linear model statistics *p*<.05 was considered statistically significant

## Discussion

The main results of this study could be summarized as follows: (1) Variable PSV was associated with marginally improved gas exchange and decreased respiratory effort when compared to BIPAP. (2) Ventilation with Variable PSV showed an association with higher amount of pulmonary edema, but did not relevantly increase overall alveolar damage and systemic or pulmonary inflammation. In contrast to our preliminary studies [14, 17, 18, 23] ventilator settings in the present trial were chosen according to clinical practice without trying to match mean airway pressure and/or mean tidal volume.

### Effects of cardiorespiratory function

Early induction of spontaneous breathing has been repeatedly shown to improve pulmonary function and attenuate pulmonary inflammation in the experimental as well as in the clinical setting [1, 11]. As a possible mechanism behind improvement of gas exchange increased pulmonary aeration especially in juxtadiaphragmal regions was discussed [24]. Our group demonstrated that redistribution of pulmonary blood flow towards better aerated lung regions, resulting in improved ventilation/perfusion matching, but not alveolar recruitment and/or increase in pulmonary aeration alone improve pulmonary gas exchange during spontaneous breathing [23]. The excellent functional variables of the respiratory system in both groups of the current study support such assumptions. However, oxygenation was probably driven to a relevant degree by the high PEEP levels used here. The approach to setting individual PEEP according to minimal elastance was chosen, as a previous study suggested spontaneous breathing with higher PEEP in experimental ARDS to better stabilize dependent lung segments and more effectively prevent from pulmonary inflammation [25]. Due to the effective lung recruitment absolute differences of gas exchange variables between experimental groups remained smaller than in our previous study [14]. However, the significant higher PaO_2_/FiO_2_ ratio and the better pH in combination with the lower PaCO_2_ seen with variable PSV indicate improved alveolar ventilation and gas exchange compared to BIPAP. The improved alveolar ventilation can largely be explained by the higher mean tidal volume and total minute ventilation generated by the assistance of breathing, while unassisted spontaneous breaths remained relatively low under BIPAP. Conceptually, some researchers consider unassisted spontaneous breaths during BIPAP to be generally irrelevant in increasing minute volume, instead emphasize their overall potential to improve cardiorespiratory function [26]. However, this view has been recently challenged by the finding, that excessive inspiratory efforts might result in lung damage and spontaneous tidal volumes and pressures need to be closely observed during respiratory therapy, instead [27]. In addition to differences in ventilation, lower mean airway pressure in the variable PSV group may have favored a redistribution of pulmonary blood flow towards better ventilated areas especially in the setting of higher PEEP, which finally resulted in an improved ventilation/perfusion matching as indicated by previous published studies [14, 17, 23].

It is worth noting that higher mean airway and lower peak airway pressures found in the BIPAP group are related to the different I: E settings (1:1 with BIPAP vs. app. 1:4 with variable PSV) because airway pressures were calculated as mean values over time. When compared to our previous study with lower PEEP and FiO_2_ settings, spontaneous respiratory rate in BIPAP was markedly lower and no signs of discomfort could be observed [14]. PTP and P_0.1_ as surrogates for respiratory workload were obviously higher with BIPAP compared to the fully assisted breaths with variable PSV. The decision if a reduction of respiratory workload in mechanically ventilated patients is desirable or not should be made in the context of each individual case. Patients running into muscle fatigue could benefit from relief of the work of breathing through fully assisted spontaneous breathing, whereas a certain amount of respiratory work by non- or partially supported spontaneous breathing could be helpful to prevent disuse atrophy of respiratory muscles in other scenarios [5].

The increase in cardiac output with BIPAP may be due to increased muscle activity also expressed by the markedly increased inspiratory effort. On the other hand, increased venous return due to a more pronounced cyclic reduction in intrathoracic pressure with BIPAP could lead to an increase in left ventricular stroke volume (SV). Left ventricular output could also be enhanced through reduction in systemic vascular resistance due to decreased pH and permissive hypercapnia with BIPAP. However, this effect is not supposed to play a major role. In addition, hyperoxia-dependent vasoregulatory mechanisms, including closure of ATP-sensitive potassium channels and increased production of endothelin-1 and 20-hydroxyeicosatetraenoic acid (20-HETE), may have contributed to increased systemic vascular resistance as seen during variable PSV.

### Effects on lung protection

In recent years, possible adverse side effects of spontaneous breathing in the acute phase of respiratory failure became apparent. Excessive efforts can generate high transpulmonary driving pressures and thereby contribute to patient self-inflicted lung injury (P-SILI) [28]. This mechanism may be particularly relevant during PSV, where negative pleural pressure swings are superimposed on ventilator-delivered pressure support. In contrast, unassisted spontaneous breaths during BIPAP/APRV lack this additional ventilator contribution and may therefore be more lung protective, provided that respiratory effort remains within a physiological range [29]. Dynamic computed tomography demonstrated reduced tidal reaeration and hyperaeration during BIPAP compared with PSV, suggesting lower cyclic lung stress despite similar end-expiratory aeration [18]. These differences were mainly attributable to the lower tidal volumes of unassisted spontaneous breaths during BIPAP. Thus, PSV may have an inherent theoretical disadvantage compared with BIPAP/APRV with regard to lung protection, independent of the addition of variability. However, this disadvantage has not translated into clinically relevant outcome differences in the limited clinical data available to date [30]. Likewise, we previously demonstrated that introducing variability into PSV does not increase pulmonary inflammation [25]. The findings of the present study are largely consistent with these considerations.

We intentionally compared both modes under conditions optimized for lung protection. The lavage model is highly recruitable, and the combination of high PEEP and FiO₂ likely stabilized lung units effectively. Consequently, overall pulmonary inflammation and histological injury were low and did not differ substantially between groups. Under such conditions, potential disadvantages of PSV may be attenuated by the relatively low vulnerability of the lung and by the modest improvements in lung function observed with PSV.

The higher degree of pulmonary edema observed during variable PSV should therefore be interpreted cautiously. One possible explanation is increased transpulmonary stress resulting from the combination of spontaneous inspiratory effort and pressure support. Differences in minute ventilation may also have contributed, as ventilatory intensity has been linked to ventilator-induced lung injury [31]. However, transpulmonary pressures could not be analyzed in the present study due to invalid esophageal pressure measurements, particularly absolute values, which became apparent in the offline analysis of some animals, rendering the above mentioned interpretations speculative. Alternatively, given the exploratory nature of the study and the number of endpoints assessed, a chance finding cannot be excluded. This interpretation is supported by the absence of corresponding differences in overall diffuse alveolar damage scores or inflammatory biomarkers, despite the observed differences in alveolar edema and wet-to-dry ratio. Although one could question the selective choice of parameters to determine lung parenchymal stress and inflammatory response in this study, the same methods yielded significant differences in previous studies employing the same experimental model [10, 11].

### Further study limitations

First, the lavage model represents a highly standardized experimental model that does not fully reflect the complex pathophysiology of clinical acute respiratory failure/ARDS, including heterogeneous inflammatory injury, edema formation, and reduced recruitability. Consequently, the transferability of our findings to the clinical setting, particularly to severe ARDS, is limited. In addition, the relatively high PEEP levels and the use of an FiO₂ of 1.0 may further restrict the generalizability of our results. Therefore, our findings should primarily be interpreted as mechanistic observations and require confirmation in more clinically representative ARDS models. Second, since minute ventilation, mean airway pressure or transpulmonary driving pressure were not matched in this protocol, we cannot exclude the possibility that the improved gas exchange observed in the variable PSV group was at least partly attributable to differences in ventilatory intensity rather than to the ventilation mode itself. Third, the absence of an experimental group with pure controlled mechanical ventilation precludes direct conclusions regarding the magnitude of spontaneous breathing-related effects in the present model. However, both ventilation modes have previously been compared with controlled ventilation in experimental studies, including studies using the same lavage-induced lung injury model [11, 32], as well as in clinical investigations [3, 33]. Finally, the modest pulmonary inflammatory response and parenchymal stress observed in both groups should be interpreted cautiously in light of the relatively short experimental duration. Consequently, the absence of relevant between-group differences does not exclude the possibility that divergent inflammatory effects may emerge over longer observation periods.

## Conclusions

In this experimental model of acute respiratory failure in pigs, variable PSV and BIPAP with non-assisted spontaneous breathing improved respiratory function. Variable PSV was marginally superior to BIPAP regarding gas exchange and was associated with reduced respiratory workload. Whereas overall lung damage and inflammatory responses were similar between groups, a detrimental effect of consequent spontaneous breath assistance during variable PSV on pulmonary edema cannot be excluded.

## Data Availability

The complete dataset of the current trial is available from the corresponding author upon reasonable request.

## Abbreviations

APRV: Airway pressure release ventilation
ARDS: Acute respiratory distress syndrome
BALF: Bronchoalveolar lavage fluid
BIPAP: Biphasic positive airway pressure
E_RS_: Elastance of the respiratory system
FiO_2_: Fraction of inspired oxygen
I:E: Inspiratory: expiratory ratio
IL: Interleukin
MPAP: Mean pulmonary arterial pressure
mRNA: Messenger ribonucleic acid
MV: Minute ventilation
P_0.1_: Airway pressure at 100 ms after beginning of inspiration
P_a_O_2_: Arterial partial pressure of oxygen
P_a_CO_2_: Arterial partial pressure of carbon dioxide
PEEP: Positive endexpiratory pressure
P_mean_: Mean airway pressure
P_peak_: Peak airway pressure
PSV: Pressure support ventilation
PTP: Pressure time product
V_T_: Tidal volume

## References

[1] Putensen C, Zech S, Wrigge H, Zinserling J, Stüber F, Von Spiegel T. u. a. Long-term effects of spontaneous breathing during ventilatory support in patients with acute lung injury. Am J Respir Crit Care Med 1 Juli. 2001;164(1):43–9. 10.1164/ajrccm.164.1. .2001078 PubMed PMID: 11435237. 11435237

[2] Putensen C, Muders T, Varelmann D, Wrigge H. The impact of spontaneous breathing during mechanical ventilation. Curr Opin Crit Care Februar. 2006;12(1):13–8. PubMed PMID: 16394778. 10.1097/01.ccx.0000198994.37319.60 16394778

[3] Zhou Y, Jin X, Lv Y, Wang P, Yang Y, Liang G. u. a. Early application of airway pressure release ventilation may reduce the duration of mechanical ventilation in acute respiratory distress syndrome. Intensive Care Med November. 2017;43(11):1648–59. 10.1007/s00134-017-4912-z . PubMed PMID: 28936695; PubMed Central PMCID: PMC5633625. PMC5633625 28936695

[4] Richard JCM, Beloncle FM, Béduneau G, Mortaza S, Ehrmann S, Diehl JL. u. a. Pressure control plus spontaneous ventilation versus volume assist-control ventilation in acute respiratory distress syndrome. A randomised clinical trial. Intensive Care Med Oktober. 2024;50(10):1647–56. 10.1007/s00134-024-07612-3 . PubMed PMID: 39287651; PubMed Central PMCID: PMC11457688. PMC11457688 39287651

[5] Levine S, Nguyen T, Taylor N, Friscia ME, Budak MT, Rothenberg P. u. a. Rapid disuse atrophy of diaphragm fibers in mechanically ventilated humans. N Engl J Med 27 März. 2008;358(13):1327–35. doi:10.1056/NEJMoa070447 PubMed PMID: 18367735. 10.1056/NEJMoa070447 18367735

[6] Suki B, Alencar AM, Sujeer MK, Lutchen KR, Collins JJ, Andrade JS. Jr, u. a. Life-support system benefits from noise. Nat 14 Mai. 1998;393(6681):127–8. doi:10.1038/30127 PubMed PMID: 9603516. 10.1038/30130 9603516

[7] Lefevre GR, Kowalski SE, Girling LG, Thiessen DB, Mutch WA. Improved arterial oxygenation after oleic acid lung injury in the pig using a computer-controlled mechanical ventilator. Am J Respir Crit Care Med November. 1996;154(5):1567–72. 10.1164/ajrccm.154.5.8912782 . PubMed PMID: 8912782. 8912782

[8] McMullen MC, Girling LG, Graham MR, Mutch WAC. Biologically variable ventilation improves oxygenation and respiratory mechanics during one-lung ventilation. Anesthesiology Juli. 2006;105(1):91–7. PubMed PMID: 16809999. 10.1097/00000542-200607000-00017 16809999

[9] Spieth PM, Carvalho AR, Güldner A, Pelosi P, Kirichuk O, Koch T. u. a. Effects of different levels of pressure support variability in experimental lung injury. Anesthesiology Februar. 2009;110(2):342–50. 10.1097/ALN.0b013e318194d06. e PubMed PMID: 19194161. 19194161

[10] Spieth PM, Carvalho AR, Pelosi P, Hoehn C, Meissner C, Kasper M. u. a. Variable tidal volumes improve lung protective ventilation strategies in experimental lung injury. Am J Respir Crit Care Med 15 April. 2009;179(8):684–93. 10.1164/rccm.200806-975. OC PubMed PMID: 19151194. 19151194

[11] Spieth PM, Carvalho AR, Güldner A, Kasper M, Schubert R, Carvalho NC. u. a. Pressure support improves oxygenation and lung protection compared to pressure-controlled ventilation and is further improved by random variation of pressure support. Crit Care Med April. 2011;39(4):746–55. 10.1097/CCM.0b013e318206bda6 . PubMed PMID: 21263322. 21263322

[12] Dos Santos Rocha A, Südy R, Bizzotto D, Kassai M, Carvalho T, Dellacà RL. u. a. Benefit of Physiologically Variable Over Pressure-Controlled Ventilation in a Model of Chronic Obstructive Pulmonary Disease: A Randomized Study. Front Physiol. 2020;11:625777. 10.3389/fphys.2020.625777 . PubMed PMID: 33519528; PubMed Central PMCID: PMC7839245. 33519528 PMC7839245

[13] Dos Santos Rocha A, Peták F, Carvalho T, Habre W, Balogh AL. Physiologically variable ventilation prevents lung function deterioration in a model of pulmonary fibrosis. J Appl Physiol Bethesda Md 1985 1 April. 2022;132(4):915–24. 10.1152/japplphysiol.00670. .2021 PubMed PMID: 35201935. 35201935

[14] Gama de Abreu M, Spieth PM, Pelosi P, Carvalho AR, Walter C, Schreiber-Ferstl. A, u. a. Noisy pressure support ventilation: a pilot study on a new assisted ventilation mode in experimental lung injury. Crit Care Med März. 2008;36(3):818–27. 3A PubMed PMID: 18431269. 10.1097/01.CCM.0000299736.55039.3A 18431269

[15] Spieth PM, Güldner A, Beda A, Carvalho N, Nowack T, Krause A. u. a. Comparative effects of proportional assist and variable pressure support ventilation on lung function and damage in experimental lung injury. Crit Care Med September. 2012;40(9):2654–61. 10.1097/CCM.0b013e3182592021 . PubMed PMID: 22743778. 22743778

[16] Spieth PM, Güldner A, Huhle R, Beda A, Bluth T, Schreiter D. u. a. Short-term effects of noisy pressure support ventilation in patients with acute hypoxemic respiratory failure. Crit Care Lond Engl 31 Oktober. 2013;17(5):R261. 10.1186/cc13091 . PubMed PMID: 24172538; PubMed Central PMCID: PMC4056040. PMC4056040 24172538

[17] Carvalho AR, Spieth PM, Pelosi P, Beda A, Lopes AJ, Neykova B. u. a. Pressure support ventilation and biphasic positive airway pressure improve oxygenation by redistribution of pulmonary blood flow. Anesth Analg September. 2009;109(3):856–65. 10.1213/ane.0b013e3181aff245 . PubMed PMID: 19690258. 19690258

[18] Gama de Abreu M, Cuevas M, Spieth PM, Carvalho AR, Hietschold V, Stroszczynski C. u. a. Regional lung aeration and ventilation during pressure support and biphasic positive airway pressure ventilation in experimental lung injury. Crit Care Lond Engl. 2010;14(2):R34. 10.1186/cc8912 . PubMed PMID: 20233399. PMC2887141 20233399

[19] Lachmann B, Robertson B, Vogel J. In vivo lung lavage as an experimental model of the respiratory distress syndrome. Acta Anaesthesiol Scand Juni. 1980;24(3):231–6. PubMed PMID: 7445941. 10.1111/j.1399-6576.1980.tb01541.x 7445941

[20] de Abreu MG, Quelhas AD, Spieth P, Bräuer G, Knels L, Kasper M. u. a. Comparative effects of vaporized perfluorohexane and partial liquid ventilation in oleic acid-induced lung injury. Anesthesiology Februar. 2006;104(2):278–89. PubMed PMID: 16436847. 10.1097/00000542-200602000-00013 16436847

[21] Peterson BT, Brooks JA, Zack AG. Use of microwave oven for determination of postmortem water volume of lungs. J Appl Physiol Juni. 1982;52(6):1661–3. 10.1152/jappl.1982.52. .6.1661 PubMed PMID: 7107478. 7107478

[22] Spieth PM, Knels L, Kasper M, Domingues Quelhas A, Wiedemann B, Lupp A. u. a. Effects of vaporized perfluorohexane and partial liquid ventilation on regional distribution of alveolar damage in experimental lung injury. Intensive Care Med Februar. 2007;33(2):308–14. 10.1007/s00134-006-0428-7 . PubMed PMID: 17091244. 17091244

[23] Carvalho AR, Spieth PM, Güldner A, Cuevas M, Carvalho NC, Beda A. u. a. Distribution of regional lung aeration and perfusion during conventional and noisy pressure support ventilation in experimental lung injury. J Appl Physiol Bethesda Md 1985 April. 2011;110(4):1083–92. 10.1152/japplphysiol.00804. .2010 PubMed PMID: 21270348. 21270348

[24] Neumann P, Wrigge H, Zinserling J, Hinz J, Maripuu E, Andersson LG. u. a. Spontaneous breathing affects the spatial ventilation and perfusion distribution during mechanical ventilatory support. Crit Care Med Mai. 2005;33(5):1090–5. PubMed PMID: 15891341. 10.1097/01.ccm.0000163226.34868.0a 15891341

[25] Kiss T, Bluth T, Braune A, Huhle R, Denz A, Herzog M. u. a. Effects of positive end-expiratory pressure and spontaneous breathing activity on regional lung inflammation in experimental acute respiratory distress syndrome. Crit Care Med April. 2019;47(4):e358–65. .0000000000003649 PubMed PMID: 30676338; PubMed Central PMCID: PMC6433156. 10.1097/CCM.0000000000003649 PMC6433156 30676338

[26] Putensen C, Wrigge H. Clinical review: Biphasic positive airway pressure and airway pressure release ventilation. Crit Care. 2004;8(6):492–7. doi:10.1186/cc2919 PubMed PMID: 15566621; PubMed Central PMCID: PMC1065046. 15566621 10.1186/cc2919 PMC1065046

[27] Yoshida T, Uchiyama A, Matsuura N, Mashimo T, Fujino Y. Spontaneous breathing during lung-protective ventilation in an experimental acute lung injury model: high transpulmonary pressure associated with strong spontaneous breathing effort may worsen lung injury. Crit Care Med Mai. 2012;40(5):1578–85. 10.1097/CCM.0b013e3182451c40 . PubMed PMID: 22430241. 22430241

[28] Brochard L, Slutsky AS, Pesenti A. Mechanical Ventilation to Minimize Progression of Lung Injury in Acute Respiratory Failure. Am J Respir Crit Care Med 15 Februar. 2017;195(4). 10.1164/rccm.201605. -1081CP PubMed PMID: 27626833. 27626833

[29] Richard JCM, Lyazidi A, Akoumianaki E, Mortaza S, Cordioli RL, Lefebvre JC. u. a. Potentially harmful effects of inspiratory synchronization during pressure preset ventilation. Intensive Care Med November. 2013;39(11):2003–10. 10.1007/s00134-013-3032-7 . PubMed PMID: 23928898. 23928898

[30] Maxwell RA, Green JM, Waldrop J, Dart BW, Smith PW, Brooks D. u. a. A randomized prospective trial of airway pressure release ventilation and low tidal volume ventilation in adult trauma patients with acute respiratory failure. J Trauma September. 2010;69(3):501–10. 10.1097/TA.0b013e3181e75961. discussion 511. 20838119

[31] Simbruner G, Mittal RA, Smith J, Maritz G, van Rensberg J, Simbruner B. u. a. Effects of duration and amount of lung stretch at biophysical, biochemical, histological, and transcriptional levels in an in vivo rabbit model of mild lung injury. Am J Perinatol März. 2007;24(3):149–59. 10.1055/s-2007-970177 . PubMed PMID: 17372857. 17372857

[32] Carvalho NC, Güldner A, Beda A, Rentzsch I, Uhlig C, Dittrich S. u. a. Higher levels of spontaneous breathing reduce lung injury in experimental moderate acute respiratory distress syndrome. Crit Care Med November. 2014;42(11):e702–715. 10.1097/CCM.0000000000000605 . PubMed PMID: 25162475. 25162475

[33] van Haren F, Pham T, Brochard L, Bellani G, Laffey J, Dres M. u. a. Spontaneous Breathing in Early Acute Respiratory Distress Syndrome: Insights From the Large Observational Study to UNderstand the Global Impact of Severe Acute Respiratory FailurE Study. Crit Care Med Februar. 2019;47(2):229–38. .0000000000003519 PubMed PMID: 30379668; PubMed Central PMCID: PMC6336491. 10.1097/CCM.0000000000003519 PMC6336491 30379668

